# Heparin Administered to *Anopheles* in Membrane Feeding Assays Blocks *Plasmodium* Development in the Mosquito

**DOI:** 10.3390/biom10081136

**Published:** 2020-08-01

**Authors:** Elena Lantero, Jessica Fernandes, Carlos Raúl Aláez-Versón, Joana Gomes, Henrique Silveira, Fatima Nogueira, Xavier Fernàndez-Busquets

**Affiliations:** 1 Institute for Bioengineering of Catalonia (IBEC), The Barcelona Institute of Science and Technology, Baldiri Reixac 10–12, ES-08028 Barcelona, Spain; elantero@ibecbarcelona.eu; 2 Barcelona Institute for Global Health (ISGlobal, Hospital Clínic-Universitat de Barcelona), Rosselló 149-153, ES-08036 Barcelona, Spain; 3 Global Health and Tropical Medicine, Instituto de Higiene e Medicina Tropical, Universidade Nova de Lisboa (IHMT-NOVA), Rua da Junqueira 100, 1349-008 Lisbon, Portugal; jessica.j.fernandes94@gmail.com (J.F.); joana.matias.gomes@gmail.com (J.G.); hsilveira@ihmt.unl.pt (H.S.); fnogueira@ihmt.unl.pt (F.N.); 4 BIOIBERICA S.A.U., Polígon Industrial “Mas Puigvert”, Ctra. N-II, km. 680.6, ES-08389 Palafolls, Spain; cralaez@bioiberica.com; 5 Nanoscience and Nanotechnology Institute (IN2UB, Universitat de Barcelona), Martí i Franquès 1, ES-08028 Barcelona, Spain

**Keywords:** malaria, heparin, mosquito, *Plasmodium*, *Anopheles*, ookinete, transmission blocking, antimalarial drugs

## Abstract

Innovative antimalarial strategies are urgently needed given the alarming evolution of resistance to every single drug developed against *Plasmodium* parasites. The sulfated glycosaminoglycan heparin has been delivered in membrane feeding assays together with *Plasmodium berghei*-infected blood to *Anopheles stephensi* mosquitoes. The transition between ookinete and oocyst pathogen stages in the mosquito has been studied in vivo through oocyst counting in dissected insect midguts, whereas ookinete interactions with heparin have been followed ex vivo by flow cytometry. Heparin interferes with the parasite’s ookinete–oocyst transition by binding ookinetes, but it does not affect fertilization. Hypersulfated heparin is a more efficient blocker of ookinete development than native heparin, significantly reducing the number of oocysts per midgut when offered to mosquitoes at 5 µg/mL in membrane feeding assays. Direct delivery of heparin to mosquitoes might represent a new antimalarial strategy of rapid implementation, since it would not require clinical trials for its immediate deployment.

## 1. Introduction

The emergence and spread of *Plasmodium falciparum* resistance to most of the existing antimalarial drugs is a key factor that contributes to the global reappearance of malaria [1]. This threat of treatment failure is prompting research oriented to targeting the transmission stages of the pathogen between humans and mosquitoes [2], represented by smaller populations less likely to contain resistant individuals that would benefit from the removal of susceptible parasites [3]. Transmission-blocking vaccines (TBV) aim at stimulating in the human the production of antibodies to actively target and block the parasite development once it is in the mosquito [4]. Among the candidate antigens for TBV strategies are proteins on the surface of the ookinete [5,6], the motile *Plasmodium* stage that forms in the blood bolus and has to traverse the midgut endothelium to progress to the next stage of the parasite, the oocyst.

Among other transmission-blocking strategies that can be envisaged is the interference with parasite–midgut interaction through the inhibitory action of the sulfated glycosaminoglycan (sGAG) heparin and related molecules, which have already shown antimalarial activity against several *Plasmodium* stages in humans. During initial malaria infection in the liver, heparin and heparan sulfate are hepatocyte receptors for sporozoite attachment [7]. In blood stages, heparin antimalarial activity, against which no resistances have been reported so far, unfolds by inhibition of merozoite invasion of the erythrocyte [8]. Chondroitin sulfate proteoglycans in the mosquito midgut and a synthetic polysulfonated polymer that mimics the structure of sGAGs present in the midgut epithelium have been described to bind *Plasmodium* ookinetes during host epithelial cell invasion [9,10], whereas ookinetes and ookinete-secreted proteins possess significant binding to heparin [11,12]. Here, we have explored the potential of heparin against ookinete development.

## 2. Materials and Methods

All reagents were purchased from Sigma-Aldrich Corporation (St. Louis, MO, USA) unless otherwise specified. Heparin from pig intestinal mucosa was provided by BIOIBERICA (Palafolls, Spain). Oversulfation to generate hypersulfated heparin was done as previously described [13], obtaining a preparation with three sulfate groups/disaccharide (as compared to 1.9–2.0 in native heparin). Briefly, 100 mg of sodium heparin salt were subjected to cation-exchange chromatography to obtain tributylamine salt, lyophilized, and dissolved in 0.8 mL of *N*,*N*-dimethylformamide, which contained an excess of pyridine-sulfur trioxide. After 1 h at 40 °C, 1.6 mL of water were added, and the product was precipitated with three volumes of cold ethanol saturated with anhydrous sodium acetate and collected by centrifugation. The product was dissolved in water, extensively dialyzed, and recovered by freeze-drying.

### 2.1. Animals

Female CD1 mice (*Mus musculus*) from the Instituto de Higiene e Medicina Tropical animal house were used to obtain blood for membrane feeding assays (MFAs) and mosquito infections, with the corresponding license (009511 from 21 April 2019) approved by the Portuguese National Authority Health (DGAV). For ex vivo production of ookinetes, female BALB/c mice (Janvier Labs, Le Genest-Saint-Isle, France) were used, following the protocols reviewed and approved by the Ethical Committee on Clinical Research from the *Hospital Clínic de Barcelona* (Reg. 10100/P2, approved on January 2018). In all cases, for the experimental procedure, mice were anesthetized using 100 mg/kg ketamine (Ketolar) mixed with 10 mg/kg xylazine (Rompun) intraperitoneally (i.p.) administered, and regularly monitored. *Anopheles stephensi* mosquitoes were maintained under standard insectary conditions (26 ± 1 °C, 75% humidity and a 12/12 h light/dark cycle). Adult mosquitoes were fed on 10% glucose solution ad libitum until the day before feeding trials.

### 2.2. Sugar Feed

Heparin directly dissolved at 5 mg/mL or 50 mg/mL in 10% glucose in H_2_O, or 10% glucose in H_2_O for the control group, were administered to mosquitoes twice (once each 24 h) in a cotton pad on the top of a net-capped paper cup containing 40–50 *A. stephensi* females. Mosquitoes were allowed to feed for 48 h, and then infected by direct feeding on a CD1 mouse parasitized with *Plasmodium berghei* ANKA-GFP (259cl1; MRA-865 [14]) for 10 min. Non-fed mosquitoes were removed, and fed mosquitoes were placed in the insectary at 21 °C and 75% humidity to allow parasite development. After eight days, mosquitoes were dissected, and the number of GFP-expressing oocysts per midgut was counted manually using an Axioskop fluorescence microscope (Zeiss, Oberkochen, Germany). The total number of mosquitoes analyzed was 61 in the control group, 63 treated with 5 mg/mL heparin, and 46 treated with 50 mg/mL heparin, distributed in three independent experiments.

### 2.3. Membrane Blood Feeding Assay

Blood obtained from an intracardiac puncture of a CD1 mouse infected with *P. berghei* ANKA-GFP (259cl1; MRA-865) was treated with 1/10 volume of 3.2% *w*/*v* sodium citrate to prevent coagulation. 6.25 µL of a solution prepared by dissolving heparin at 40 mg/mL or 0.4 mg/mL in phosphate buffered saline (PBS) was added to 494 µL of blood:citrate (to obtain final heparin concentrations of 500 and 5 µg/mL, respectively), which was then placed in feeders prepared with two-sided stretched Parafilm^®^ connected to two plastic tubes for water inlet and outlet. The same volumes of PBS and blood:citrate were used for heparin-free controls. Temperature within the multiple cylindrical water-jacked glass was kept at 37 °C by a constant water flow supply. Each feeder was placed on top of a net-covered paper cup containing 40–50 *A. stephensi* females. Mosquitoes were allowed to feed for one hour. Non-fed mosquitoes were removed, and the rest were treated as above. The final number of mosquitoes analyzed for the non-modified heparin assay was 127 in the control group, 106 treated with 5 µg/mL heparin, and 149 treated with 500 µg/mL heparin, distributed in three independent experiments. The final number of mosquitoes analyzed for the hypersulfated heparin assay was 62 in the control group, 91 treated with 5 µg/mL hypersulfated heparin, and 102 treated with 500 µg/mL hypersulfated heparin, distributed in two independent experiments.

### 2.4. Detection of Heparin-Cy5 in Mosquitoes

Thirty female *A. stephensi* mosquitoes per cage were allowed to feed on 400 µg/mL heparin-Cy5 (Nanocs Inc., New York, NY, USA) on either sugar for 6 h or MFA for 1 h. Some mosquitoes were taken from the cages at 6, 24, 48, and 72 h post-feeding to check Cy5 fluorescence (λ_ex_/λ_em_: 650/670 nm) with an Eclipse 80i microscope (Nikon, Tokyo, Japan). Mosquitoes with fluorescent signal were dissected, and their organs were individually observed.

### 2.5. Ex Vivo Production of Ookinetes and Flow Cytometry Analysis

Eight days before ookinete production, 200 µL of *P. berghei* CTRP-GFP (kindly provided by Dr. Inga Siden-Kiamos [15]) in cryopreservation solution (RBC pellet:Roswell Park Memorial Institute medium (RPMI, Gibco, Dublin, Ireland):30% glycerol in water, 1:1:2) was administered i.p. to a BALB/c mouse. Four days later, this mouse was the donor to infect i.p. with 5 × 10^7^ parasitized red blood cells in 200 µL of PBS a second mouse that one hour before the infection had been pretreated i.p. with phenylhydrazine (120 µL of a 10 mg/mL solution in PBS). For ookinete production, up to 1 mL of blood carrying gametocytes was collected by intracardiac puncture and diluted in 30 mL of ookinete medium: 10.4 g/L of RPMI supplemented with 2% *w*/*v* NaHCO_3_, 0.05% *w*/*v* hypoxanthine, 0.02% *w*/*v* xanthurenic acid, 50 U/mL penicillin and 50 µg/mL streptomycin, 20% heat-inactivated fetal bovine serum (FBS, Invitrogen, Carlsbad, CA, USA), 25 mM HEPES, pH 7.4. The culture was incubated for 24 h at 21 °C with orbital shaking at 50 rpm (modified from [16]).

To check heparin influence on fertilization, to 487.5 µL of culture in a well of 24-well plates was added 12.5 µL of PBS containing heparin at 20 or 0.2 mg/mL, to provide final heparin concentrations of 500 µg/mL and 5 µg/mL. Samples were taken at two different time points (just after extraction and after 1 h incubation), including a control consisting of PBS only, in three independent experimental replicates. Twenty-four hours later, samples were diluted 1:100 in PBS and analyzed in a LSRFortessa^TM^ flow cytometer (BD Biosciences, San Jose, CA, USA) set up with the five lasers, 20 parameters standard configuration. The GFP positive ookinete population was selected and counted using 488 nm laser excitation and a 525/40 nm emission collection filter. BD FACSDiva software version 6.1.3 (BD Biosciences) was used in data collection, and Flowing Software 2.5.1 (Turku Centre for Biotechnology, Turku, Finland) was used for analysis.

For targeting assays, mature ookinetes were washed twice with PBS and incubated with heparin-Cy5 at 400 µg/mL in ookinete medium without FBS for 1 h. The sample was finally diluted 1:100 in the same medium containing 0.2 µg/mL Hoechst 33342 and events recorded with an Amnis^®^ ImageStream^®X^ Mk II cytometer (Luminex Corporation, Austin, TX, USA) using 375 nm, 488 nm, and 642 nm excitation lasers for Hoechst 33342, GFP and Cy5 signals respectively. Data were analyzed with IDEAS^®^ 6.3 software (Luminex Corporation).

### 2.6. Statistical Analysis

Oocysts/midgut counts from three independent experiments were plotted and analyzed in GraphPad Prism 6 using an unpaired Mann–Whitney test to determine significant differences. *t*-Tests with Welch’s correction were applied for determining significance in ex vivo ookinete maturation and targeting assays, and degrees of freedom were automatically defined by the software, according to n. In both cases, tests were two-sided.

## 3. Results

### 3.1. Characterization of Heparin-Cy5 Binding to Ookinetes

**Figure 1:**
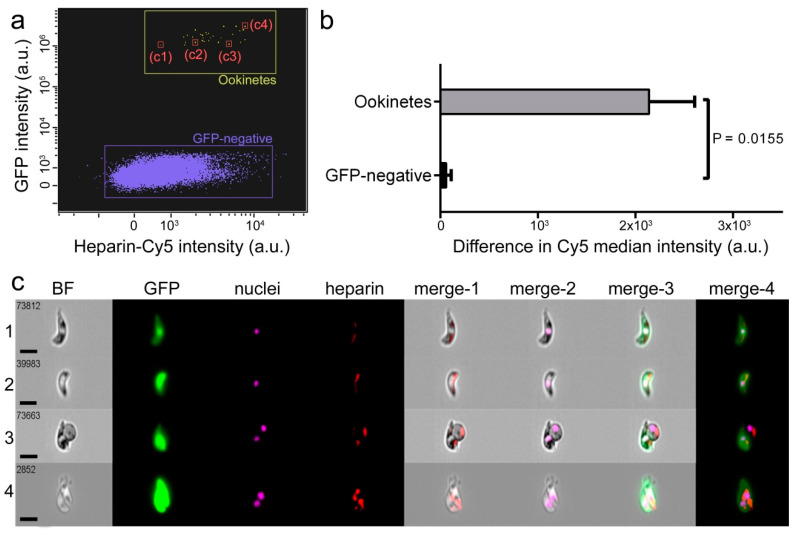
Heparin-Cy5 binding to ookinetes. (**a**) Flow cytometry plot showing heparin-Cy5 signal in green fluorescent protein (GFP)-expressing *P. berghei* ookinetes. GFP-negative events correspond mostly to red blood cells (see Figure S1 for gating strategy). c1 to c4 refer to the individual events reported in panel **c**. (**b**) Difference in Cy5 median intensity between GFP-expressing ookinetes and GFP-negative cells. (**c**) Fluorescence images of the flow cytometry events indicated in panel **a**. The merges of the bright field (BF) image with the fluorescence of heparin, nuclei, GFP, and all three of them, are indicated as merge-1 to merge-4, respectively. Size bars represent 7 µm.

Flow cytometry analysis showed binding of heparin-Cy5 to ookinetes obtained ex vivo from mouse blood infected with the *P. berghei* CTRP-GFP transgenic line (Figure 1a,b), which expresses GFP when reaching ookinete stage. Fluorescence images indicated the binding of heparin-Cy5 to discrete areas on the ookinete (Figure 1c), which suggests clustering of heparin receptors.

### 3.2. Effect on Oocyst Development of Heparin Administered to Mosquitoes by Sugar Meal

**Figure 2:**
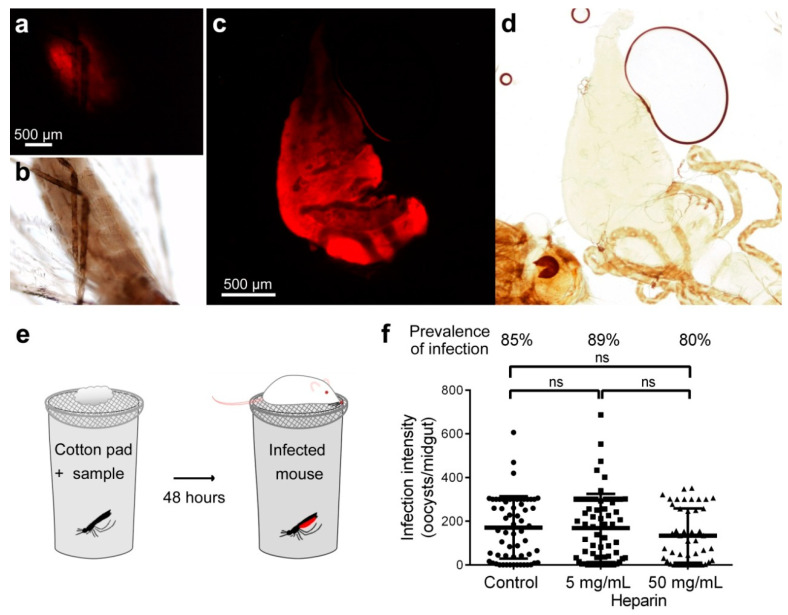
Effect on ookinete development of heparin fed to mosquitoes by sugar meal. (**a**,**c**) Fluorescence detection in (**a**) intact abdomen and (**c**) dissected midgut of heparin-Cy5 fed to *A. stephensi* female mosquitoes in a sugar meal. (**b**,**d**) Bright field images of the microscope fields in panels **a** and **c**, respectively. (**e**) Depiction of the method for sugar feed used in mosquito assays. (**f**) Effect on parasite development of heparin delivered by sugar swaps. ns: not significant.

Heparin-Cy5 fed in the sugar meal to female *A. stephensi* mosquitoes was detected in the midgut of the insects for up to 72 h after administration (Figure 2a–d and Figure S2). Heparin effect on ookinete to oocyst transition was then assessed in live mosquitoes, by offering them heparin by sugar feed during 48 h before infecting them by direct bite to a *P. berghei* ANKA-GFP-parasitized mouse (Figure 2e). Unfed mosquitoes were removed, and eight days later, mosquitoes were dissected, and GFP-expressing oocysts were counted. The prevalence of infection (PI, percentage of mosquitoes with ≥1 oocyst) and the infection intensity (II, number of oocysts per midgut) were not significantly affected when compared to untreated controls up to heparin concentrations in the sugar feed of 50 mg/mL (Figure 2f). It is likely that most of the sugar feed might be pushed out by the blood meal, in which case heparin would not interact with ookinetes. This result led us to explore new strategies to ensure the presence of heparin at the moment of ookinete development in the mosquito midgut by including heparin in a *Plasmodium*-infected blood meal.

### 3.3. Effect on Oocyst Development of Heparin Administered to Mosquitoes by Blood Membrane Feeding

**Figure 3:**
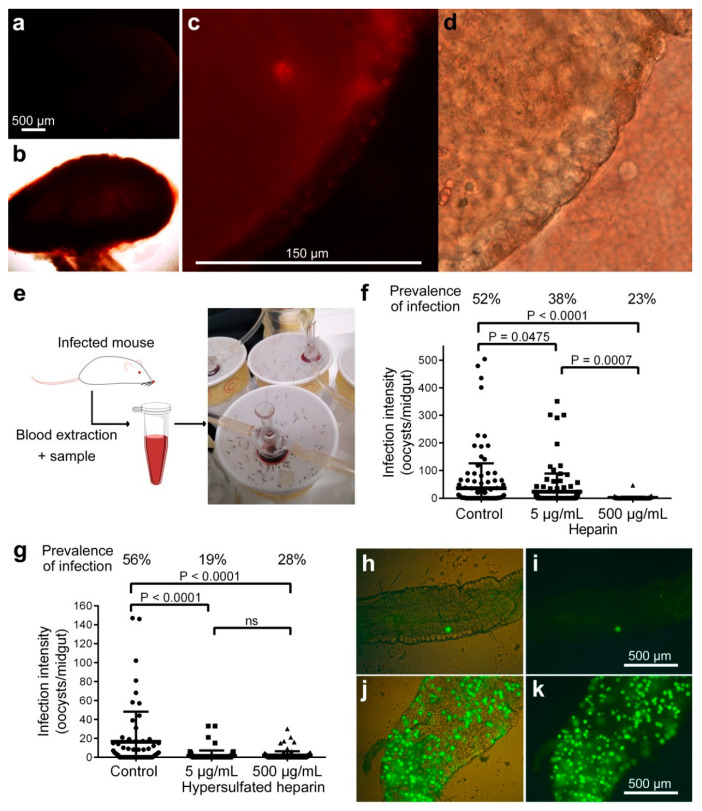
Effect on ookinete development of heparin fed to mosquitoes by membrane feeding assay (MFA). (**a**–**d**) Fluorescence detection of heparin-Cy5 fed to *A. stephensi* female mosquitoes by blood feed. (**a**,**b**) Whole dissected midgut and (**c**,**d**) magnification of the same midgut with the blood bolus pushed away. (**b**,**d**) Bright field images of the microscope fields in panels **a** and **c**, respectively. (**e**) Depiction of the MFA method used in mosquito assays. (**f**) Effect on parasite development of non-modified heparin delivered by MFA. (**g**) Effect on parasite development of hypersulfated heparin delivered by MFA. ns: not significant. (**h**–**k**) Fluorescence images of representative mosquito midguts from the MFA 500 µg/mL non-modified heparin group (**h**,**i**) and from the MFA control group (**j**,**k**); the fluorescence signal is shown alone (**i**,**k**) and merged with bright field images of the midgut contours (**h**,**j**).

Heparin-Cy5 fed to female *A. stephensi* mosquitoes by whole blood MFAs was detected in the midgut of the insect for at least 24 h after administration (Figure 3a–d and Figure S3). Often, Cy5 fluorescence was only faintly observed in the dissected midgut (Figure 3a,b), but it intensified after having pushed out the blood bolus (Figure 3c,d). This might result from light being absorbed or screened by the compacted blood bolus. Heparin activity on ookinete to oocyst transition was then assessed with this method of administration. Heparin was added to the blood of mice infected with *P. berghei* ANKA-GFP, which was then offered to female *A. stephensi* by MFA (Figure 3e). Unfed mosquitoes were removed, and eight days later, mosquitoes were dissected, and oocysts were counted. A significant decrease of PI and II was observed in mosquitoes fed with heparin-containing infected blood samples (Figure 3f,h–k). PI was 38% and 23% for respective MFA heparin concentrations of 5 µg/mL and 500 µg/mL, compared to 52% for the heparin-free control, whereas the mean II for the same samples was, respectively, 24.22 ± 65.10, 0.95 ± 4.12, and 36.38 ± 89.57 oocysts per midgut.

When a modified heparin with higher proportion of sulfated residues in the polysaccharide chain (hypersulfated heparin) was offered to mosquitoes by MFA, a significant decrease in PI and II was observed with as little as 5 µg/mL of heparin (Figure 3g). PI was 19% and 28% for respective hypersulfated heparin concentrations of 5 µg/mL and 500 µg/mL compared to 56% for the control, whereas the mean II for the same samples was, respectively, 1.53 ± 5.56, 1.74 ± 4.61, and 16.29 ± 31.94 oocysts per midgut. Although the extensive dialysis performed at the end of the heparin sulfation process should have removed any residual byproduct, future research has to rule out potential interferences of trace chemicals on the mechanism of oocyst formation. No impact on mosquito viability was observed for any of the heparins studied here (data not shown).

The observation that PI in blood feeding assays was significantly lower than in sugar meal experiments might be explained by the presence of sodium citrate, which is a calcium chelator used to prevent blood coagulation. Since the induction of exflagellation in *Plasmodium* requires calcium [17], sodium citrate could have a synergistic effect with heparin potentiating its inhibitory effect on oocyst formation. Heparin is also a potent calcium chelator which binds ca. one Ca^2+^ ion per average disaccharide [18]. At the high 50 mg/mL heparin concentration of sugar meal assays, the calcium binding capacity of heparin was comparable to that of the sodium citrate amount used in MFAs. Although in these experiments no effect of heparin was seen on ookinete development, the suspected immiscibility of sugar feed and blood meal calls for caution before drawing any conclusions regarding the suspected inhibitory effect of calcium chelators on *Plasmodium* development in the mosquito.

### 3.4. Effect of Heparin on Fertilization

**Figure 4:**
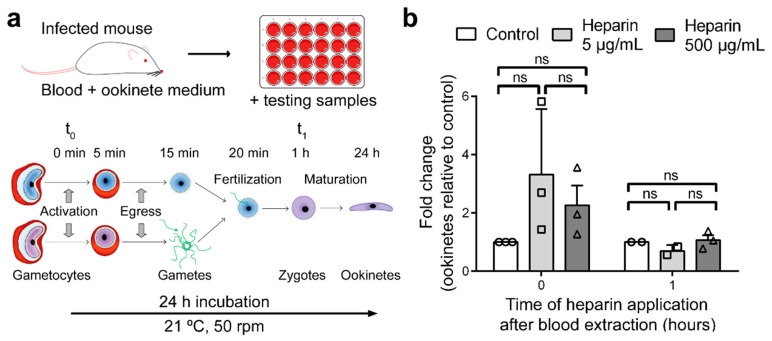
Effect of heparin in the ex vivo development of ookinetes. (**a**) Depiction of the method used for the ex vivo growth of ookinetes. The parasite development scheme has been adapted from Kuehn and Pradel [20]. (**b**) Effect of heparin on ex vivo ookinete maturation analyzed by flow cytometry (see Figure S4 and Table S1). ns: not significant.

When blood from *P. berghei*-infected mice was put into culture to obtain ookinetes and heparin was added to the culture either at the moment of blood extraction (t_0_) or 1 h later (t_1_), no significant effect was observed in the number of ookinetes produced when compared with untreated control cultures (Figure 4). However, the fold-increase in ookinete numbers when heparin was added at t_0_ (ookinetes relative to normalized control: 3.32 ± 2.26 and 2.26 ± 1.18 for 5 µg/mL and 500 µg/mL heparin, respectively), though non-significant due to the high dispersion of the results, could indicate that heparin enhances fertilization. This effect might operate through heparin interactions with coagulation factors, which would facilitate gamete motility. Heparin use in MFAs was previously recommended over other anticoagulants such as EDTA, as better infection rates were obtained [19]. Consistently, no effect on ookinete numbers was observed when heparin was added at t_1_, when fertilization has already occurred [20]. These results indicated that the inhibitory activity of heparin on *Plasmodium* mosquito stages is not exerted during fertilization or zygote maturation. Although sodium citrate was not used in ex vivo assays, the presence of 500 µg/mL heparin bound a significant amount of calcium, and yet, exflagellation was not affected. However, the potential role of calcium sequestration on this part of the parasite’s development deserves further exploration.

## 4. Discussion

The results presented above validate a potential new antimalarial strategy where heparin binding to ookinetes will prevent the interaction of this *Plasmodium* stage with the mosquito midgut and consequently its development into an oocyst. It has been suggested that chondroitin sulfate is a ligand for the circumsporozoite- and thrombospondin-related anonymous protein-related protein (CTRP) [9], a key molecule for ookinete mobility and parasite development [21]. Characterizing the sGAG ookinete binding domain and sulfation pattern will be important regarding the development of future antimalarials acting on this stage of the parasite’s cycle. To start unraveling the relevance of the sulfation pattern in blocking ookinete progression, hypersulfated heparin has been tested here, and has shown interesting potential since the lowest concentration used resulted in a significantly larger inhibition of ookinete development than the same concentration of native heparin. Chemical modifications of heparin or its binding to nanocarriers are strategies that could contribute to increase activity and midgut residence time in view of the potential development of sGAG-based antimalarials as disruptors of the life cycle of *Plasmodium* in the mosquito.

So far, transmission-blocking approaches have focused on the concept of treating humans with vaccines or drugs that will target mosquito stages [22]. A largely unexplored avenue, however, is targeting *Plasmodium* in the insect vector directly [23]. The implementation of antimalarial medicines designed to be delivered directly to mosquitoes might reduce treatment and development costs because the clinical trials otherwise required for therapies to be administered to people could be significantly simplified. Strategies that control malaria using direct action against *Anopheles* are not new but mostly focus on eliminating the vector, either by killing it with pesticides [24] or through the release of sterile males [25]. The administration of drugs to mosquitoes during their blood feed is being used to deliver ivermectin, an endectocide that, at concentrations found in human blood after treatment, is toxic to all *Anopheles* species examined [24]. When *P. falciparum*–infected female *Anopheles gambiae* mosquitoes were exposed to surfaces treated with the antimalarial drug atovaquone, the development of the parasite was completely arrested [26]. Although this strategy is unlikely to work for large hydrophilic molecules like heparin, several other approaches are available for direct drug delivery to mosquitoes, some of which have been used to deliver to dipterans lipid-based [27] and chitosan nanoparticles [28].

The failure of heparin in inhibiting parasite development when delivered in a sugar meal prior to infection of the mosquito indicates that heparin must be present in the midgut simultaneously with ookinetes. This poses a significant obstacle regarding the practical implementation of future antimalarial strategies based on the observations reported here. Heparin is normally present in human plasma in values ranging from 1 mg/L to 2.4 mg/L [29], whereas heparin in the mosquito midgut is active at concentrations >100 times higher. The anticoagulant activity of heparin prevents its administration to people in the amounts required to block ookinete development, although sGAG mimetics [9] or modified heparins having low anticoagulant capacity [30] offer promising perspectives. The use of limited heparin amounts in infected patients for transmission-blocking might actually be beneficial given the described pro-coagulant effects of *Plasmodium*-infected red blood cells [31]. However, to be present in the circulation at the moment of a mosquito bite, the blood residence time of these molecules should be extremely long. An alternative approach could be provided by offering heparin to mosquitoes in an artificial diet simulating vertebrate blood. The available technology is capable of manufacturing artificial blood for mosquito feeding from hemoglobin obtained from citrated rabbit blood [32], outdated bovine blood [33], and other blood-free artificial liquid diets [34,35,36]. These substitutes mimic in the mosquito the physiological effects of a fresh vertebrate blood meal, supporting ovarian and egg maturation and normal development of offspring into functional adults. Such artificial feedings can substitute direct feeding on mammals and often have prolonged shelf life and do not require refrigeration. For their delivery to mosquitoes, a number of artificial blood feeders are currently under study [37]. This approach would require the presence of attractants in the artificial diet to lure mosquitoes that have already taken a human blood meal and thus potentially carrying ookinetes in their midguts.

The economic landscape of malaria calls for new strategies that take into account the costs of bringing a medicine into the market, which due to expensive clinical trials often prevent promising new drugs from a fast entry into the production pipeline. As a possible approach to solving this problem, the administration of heparin to mosquitoes offers two advantages: first, blocking the life cycle of *Plasmodium* in the mosquito vector through direct drug delivery to the insect, can dramatically shorten product development due to the avoidance of large-scale human tests. Second, applying the three Rs of drug development (rescue, repurpose, reposition) to previously discarded compounds is an interesting strategy to return value to potential treatments in decline or on hold. Heparin is a natural polysaccharide that can be abundantly obtained in large amounts from the intestinal mammalian mucosa and which has a widespread medical use. The results presented here can inspire researchers and entrepreneurs, especially those in malaria endemic regions, to pursue the development of an efficient and economically affordable antimalarial strategy. A chain of heparin production could easily start with the usually discarded mucosae of pigs, goats, sheep, or cows that are consumed for food. In addition, potential strategies to deliver heparin to mosquitoes might involve the use of small containers filled with mosquito attractants, which can boost the economy of many developing regions through either the fabrication of such dispensers made of plastic, glass or aluminum, or the recycling of bottles and cans.

## Supplementary Materials

The following are available online at https://www.mdpi.com/2218-273X/10/8/1136/s1, Figure S1. Gating strategy for Figure 1a; Figure S2. Photomicrograph gallery of different time points after heparin-Cy5 administration in sugar feed; Figure S3. Photomicrograph gallery of different time points after heparin-Cy5 administration in MFA; Figure S4. Gating strategy for the data analysis presented in Figure 4b; Table S1. GFP-positive events (n) and corresponding percentages presented in Figure 4b.
