# Supplementary material for: Heparin Administered to Anopheles in Membrane Feeding Assays Blocks Plasmodium Development in the Mosquito
Source: Biomolecules. 2020 Aug 1;10(8):1136. doi: 10.3390/biom10081136 (PMC7463908; doi:10.3390/biom10081136)
Supplement: Supplementary file 1 [file biomolecules-10-01136-s001.pdf]

Heparin Administered to *Anopheles* in Membrane Feeding Assays Blocks *Plasmodium* Development in the Mosquito

Supplementary Material

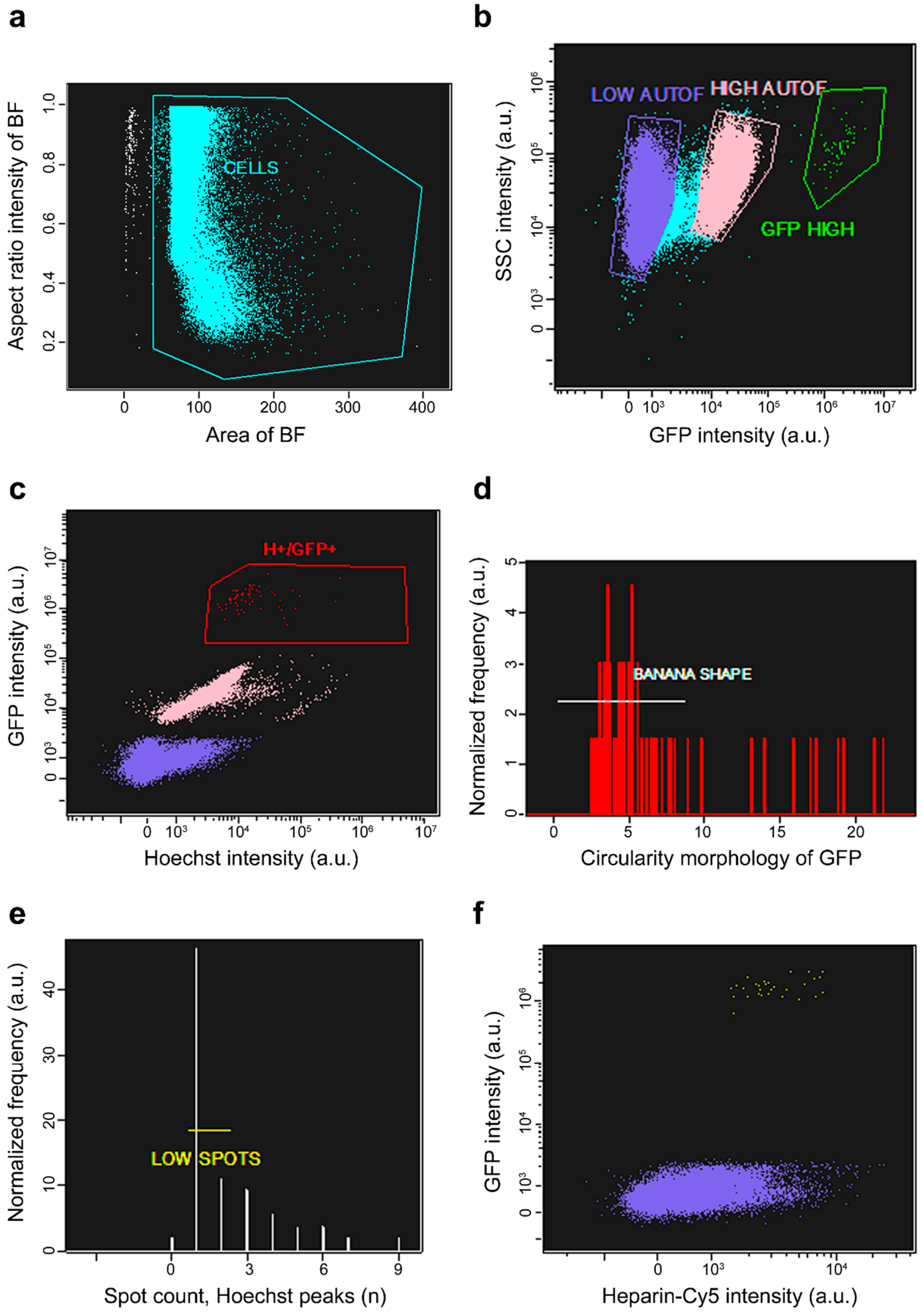

Figure S1. Gating strategy for Figure 1a. (**a**) in a dot plot of Aspect ratio intensity vs. Area of the bright field (BF) images of all events, a cell population was selected (CELLS). (**b**) in a dot plot of side scatter (SSC) intensity against GFP intensity, of the selected CELLS population, three different subgroups were defined: LOW AUTOFLUORESCENCE, HIGH AUTOFLUORESCENCE, and GFP HIGH. (**c**) Again plotting the CELLS population, the HIGH

AUTOFLUORESCENCE subgroup was confirmed to be also autofluorescent in the Hoechst channel. The H+/GFP+ subgroup corresponds to GFP HIGH in panel **b**. (**d**) To select mature ookinetes, the H+/GFP+ subgroup was analysed using a circularity morphology mask from IDEAS® software, selecting elongated, or BANANA SHAPE, cells, indicative of mature ookinetes. (**e**) using the BANANA SHAPE population, the number of Hoechst peaks was analysed with a spot count mask, where the cells with the lowest number of peaks are the LOW SPOTS population. (**f**) LOW SPOTS were mature non-aggregated ookinetes in the sample, plotted together with the LOW AUTOFLUORESCENCE subgroup for comparison. The differences in Cy5 intensity between stained and non-stained samples were then analyzed. The HIGH AUTOFLUORESCENCE subgroup had a similar difference in Cy5 intensity as the LOW AUTOFLUORESCENCE subgroup, but only this one was plotted to facilitate data interpretation. LOW SPOTS subgroup had n of 23, 8 and 6 in the 3 experimental samples, and 5, 3 and 4 in the 3 controls. LOW AUTOFLUORESCENCE subgroup had n of 37258, 47999 and 34332 in the 3 experimental samples and 14819 and 11681 in the controls.

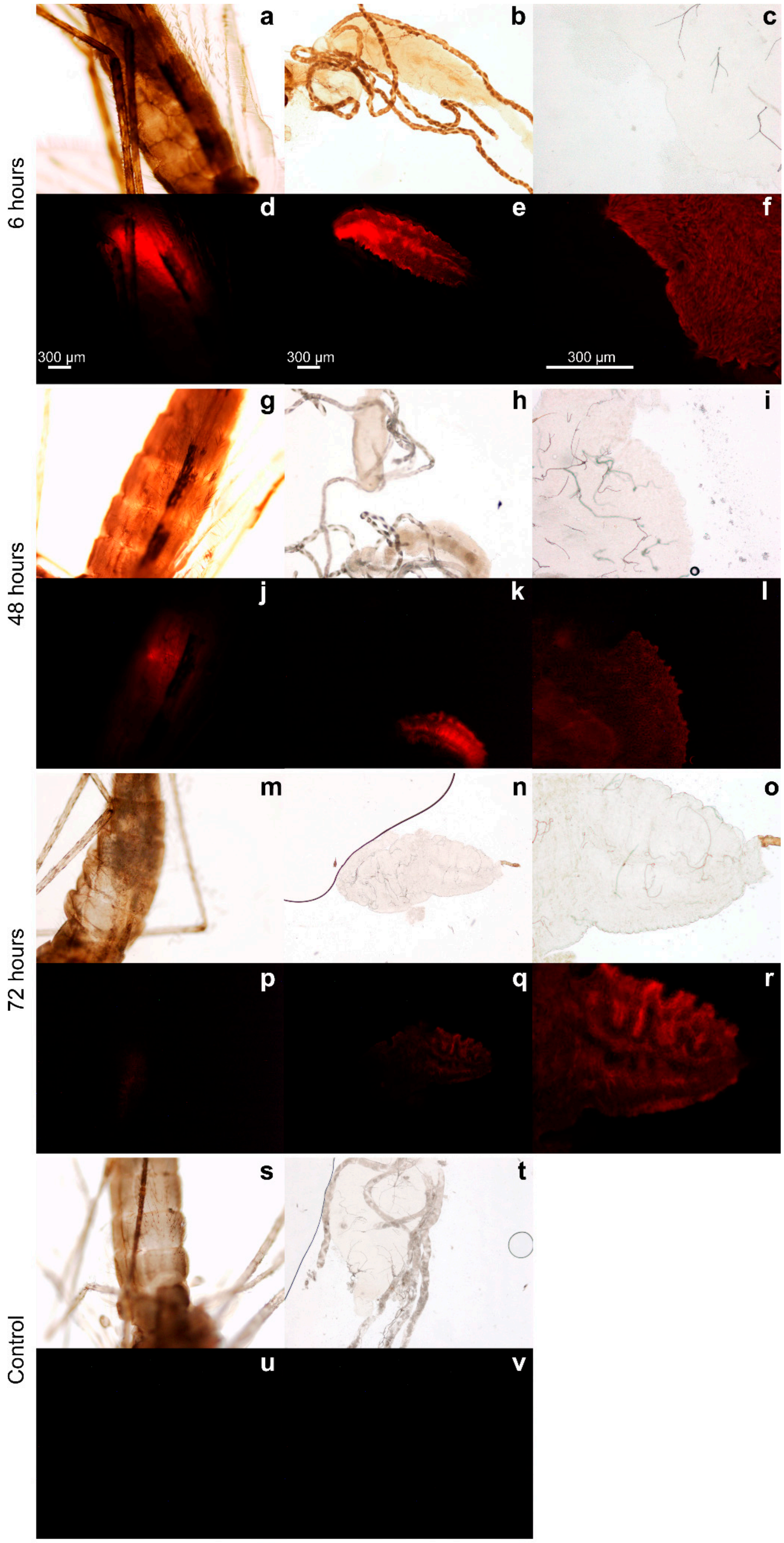

a
b
c
6 hours
d
e
f
300 µm
300 µm
300 µm
g
h
i
48 hours
j
k
l
m
n
o
72 hours
p
q
r
s
t
Control
u
v

Figure S2. Photomicrograph gallery of different time points after heparin-Cy5 administration in sugar feed. (**a**-**c**, **g**-**i**, **m**-**o**) Bright field images of mosquito abdomens (**a**,**g**,**m**), dissected midguts (**b**,**h**,**n**) and magnified images of dissected midguts (**c**,**i**,**o**), taken at 6 (**a**-**c**), 48 (**g**-**i**), and 72 h after administration (**m**-**o**). (**d**-**f**, **j**-**l**, **p**-**r**) Below each photomicrograph is the Cy5 fluorescence image of the same region. The non-fluorescent midgut in **h** comes from a non-fed mosquito. (**s**-**v**) Bright field images of the abdomen (**s**), and dissected midgut (**t**) of a sugar-only-fed control mosquito taken at 6 h after administration, and fluorescence images of the same regions (**u**,**v**, respectively) in the Cy5 emission channel (autofluorescence control).

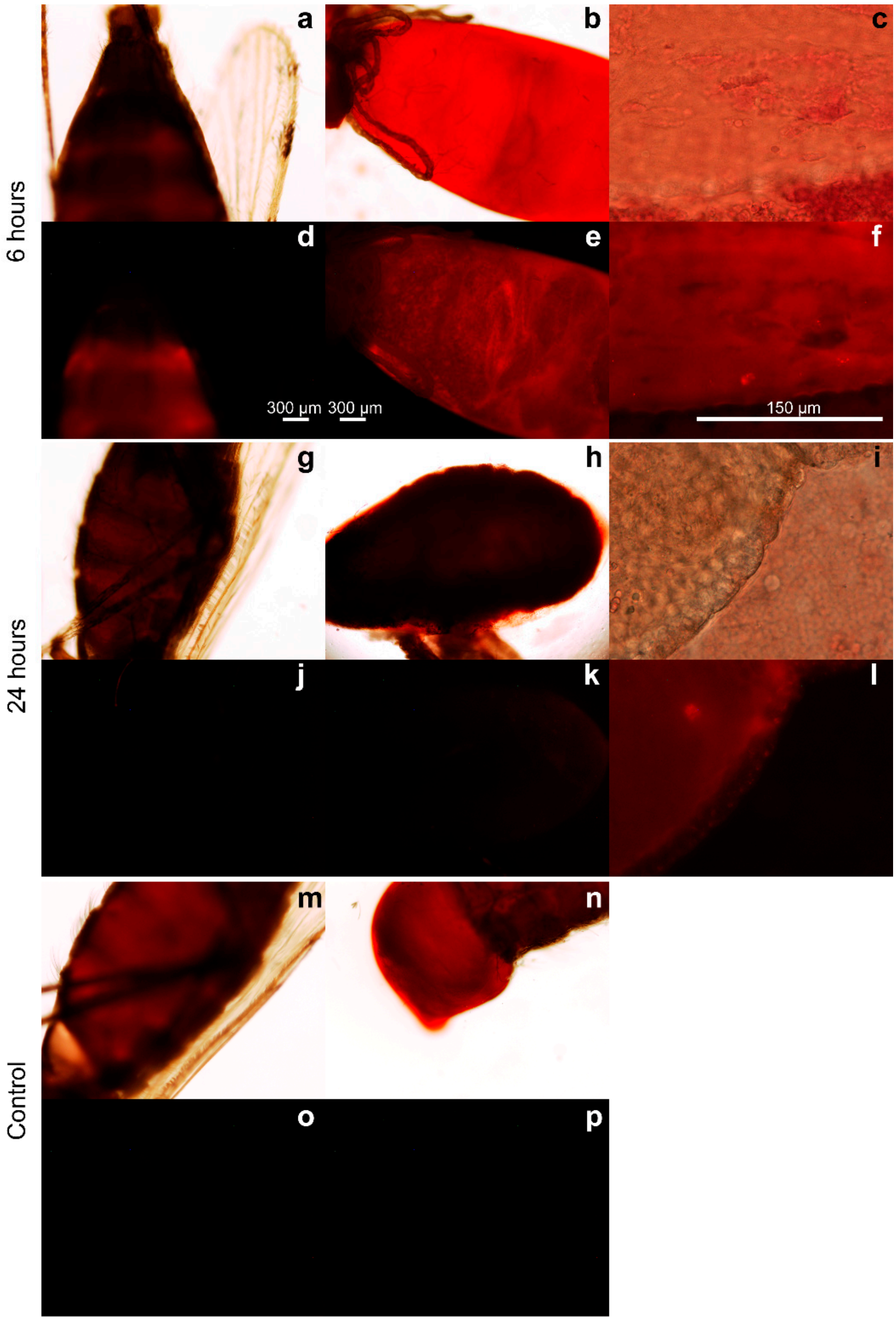

Figure S3. Photomicrograph gallery of different time points after heparin-Cy5 administration in MFA. (**a**-**c**, **g**-**i**) Bright field images of mosquito abdomens (**a**,**g**), dissected midguts (**b**,**h**) and magnified images of dissected midguts after having pushed out the blood bolus (**c**,**i**), taken at 6 (**a**-**c**) and 24 h after administration (**g**-**i**). (**d**-**f**, **j**-**l**) Below each photomicrograph is the Cy5 fluorescence image of the same region. Occasionally, Cy5 fluorescence was not observed in the intact abdomen (**j**), and only faintly in the dissected midgut (**k**), but it intensified after having pushed out the blood bolus (**l**). This might result from light being absorbed or screened by the compacted blood bolus. (**m**,**n**) Bright field images of the abdomen (**m**), and partially dissected midgut (**n**) of a blood-only-fed control mosquito taken at 6 h after administration. (**o**,**p**) Fluorescence images of the same regions in the Cy5 emission channel (autofluorescence control).

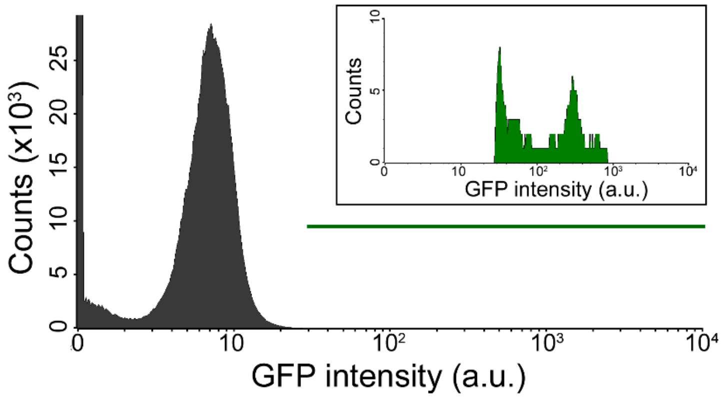

Figure S4. Gating strategy for the data analysis presented in Figure 4b. All the events were analysed in a histogram according to their GFP intensity, and cells were considered GFP-positive when their fluorescence was in the region indicated with a green line. The inset contains a histogram showing an example of GFP-positive events.

Table S1. GFP-positive events (n) and corresponding percentages presented in Figure 4b. Percentages were used for calculating fold change relative to the control in each experiment.

Time (h)	Sample	Replicate	Experiment 1		Experiment 2		Experiment 3	
			GFP-positive events	% of GFP-positive	GFP-positive events	% of GFP-positive	GFP-positive events	% of GFP-positive
0	Heparin 5 µg/mL	1	280	0.0151	462	0.1064	645	0.1381
		2	293	0.0144	458	0.0988	457	0.1142
		3	290	0.0195	465	0.0983	456	0.1114
	Heparin 500 µg/mL	1	262	0.0124	458	0.0753	459	0.0871
		2	278	0.0176	464	0.0789	460	0.0708
		3	283	0.0129	456	0.0659	461	0.0648
	Control	1	193	0.0087	407	0.0240	388	0.0174
		2	212	0.0117	458	0.0420	461	0.0219
		3	303	0.0136	459	0.0468	455	0.0232
1	Heparin 5 µg/mL	1	-	-	357	0.0160	78	0.0035
		2	-	-	290	0.0130	82	0.0041
	Heparin 500 µg/mL	1	268	0.0148	376	0.0169	117	0.0053
		2	277	0.0161	407	0.0198	117	0.0053
		3	275	0.0158	-	-	-	-
	Control	1	-	-	458	0.0215	139	0.0062
		2	-	-	267	0.0133	145	0.0072
